# Syndrome de Guillain-Barré en milieu hospitalier au Togo

**DOI:** 10.48327/mtsibulletin.2021.124

**Published:** 2021-09-04

**Authors:** K. Apetse, J.J. Dongmo Tajeuna, V.K. Kumako, K.P. Waklatsi, D. Kombate, K. Assogba, A.K. Balogou

**Affiliations:** 1 Faculté des sciences de la santé, Université de Lomé, Lomé, Togo; 2 Service de neurologie, CHU CAMPUS de Lomé, Lomé, Togo; 3 Faculté des sciences de la santé, Université de Kara, Kara, Togo; 4 Service de neurologie, CHU Kara, Kara, Togo

**Keywords:** Syndrome de Guillain-Barré, Épidémiologie, Pronostic, Hôpital, Lomé, Togo, Afrique subsaharienne, Guillain-Barré syndrome, Epidemiology, Prognosis, Hospital, Lomé, Togo, Sub-Saharan Africa

## Abstract

**Introduction:**

Les études sur le syndrome de Guillain-Barré (SGB) sont rares en Afrique subsaharienne caractérisée par un plateau technique médical limité et une fréquence relativement plus élevée de maladies infectieuses.

**Objectifs:**

Les objectifs de ce travail sont de décrire les caractéristiques du SGB au Togo à travers une cohorte de patients suivis dans le service de neurologie du CHU Campus de Lomé.

**Méthodologie:**

L’étude s'est déroulée de mai 2015 à juillet 2019. Les patients présentant un SGB des niveaux 1, 2 ou 3 des critères de certitude diagnostique de Brighton, ont été inclus de façon consécutive. Ils ont été évalués à l'admission puis à 6 mois et à 1 an d’évolution à l'aide du score d'invalidité SGB (GBS score) et du score neuromusculaire du Medical Research Council (MRC sum-score). Les variables qualitatives et quantitatives ont été exprimées, respectivement, en fréquence et en médiane (intervalle interquartile).

**Résultats:**

Sur 7 012 patients hospitalisés, 28 (0,39%) présentaient un SGB avec une prédominance féminine (20 femmes soit 71%) et un âge médian de 40 (IQ: 27-53) ans. La présentation clinique majoritairement était constituée par des troubles sensitivo-moteurs bilatéraux des membres à prédominance crurale associés à une diplégie faciale et précédés d'un évènement infectieux. A l'admission, la marche était possible (GBS score 0 à 3) chez 11 patients (39%) et le MRC sum score médian était de 28 (IQ: 12-38). La dissociation albumino-cytologique était présente chez 13 des 20 patients ayant eu une ponction lombaire (65%). Les sous-types démyélinisant et axonal ont représenté chacun 47% (9/19 patients ayant eu un électroneuromyogramme). Les immunoglobulines et la corticothérapie par voie intraveineuse ont été respectivement administrées chez 18% (n=5) et 50% (n=14) des patients. La létalité hospitalière était de 11% (n=3). Le MRC sum score médian à six et 12 mois étaient respectivement de 40 (IQ: 38-49) et 51 (IQ: 46-58). À un an d’évolution, la létalité était de 18% (n=5) et 78% (n=14) des 18 survivants pouvaient marcher sans aide dont 17% (n=3) asymptomatiques.

**Conclusion:**

Au Togo, avec une prévalence hospitalière faible, le SGB reste une affection grave de par sa forte morbi-létalité en lien avec des thérapeutiques non optimales mais aussi un retard de prise en charge.

## Abstract (en)

**Introduction:**

In sub-Saharan Africa characterized by limited resources especially in health facilities and a relatively higher frequency of infectious diseases, studies on Guillain-Barré syndrome (GBS) are rare.

**Objectives:**

The objectives of this work are to describe the characteristics of GBS in Togo through a cohort of patients followed in the neurology unit of the Campus University Hospital of Lomé.

**Methodology:**

The study took place from May 2015 to July 2019. Patients with GBS of levels 1 to 3 of the Brighton criteria for diagnostic certainty were included consecutively and assessed at admission, at 6 months and at 1 year with the GBS disability score and the MRC sum score. Qualitative and quantitative variables were expressed, respectively, in frequency and median (interquartile range).

**Results:**

Out of 7012 hospitalized patients, 28 (0.39%) including 20 women (71%) with a median age of 40 (27-53) years, presented GBS. The clinical presentation mainly consisted of bilateral sensory-motor disorders predominantly in the lower limbs associated with facial diplegia and preceded by an infectious event. On admission, 39% of patients (n=11) were able to walk (GBS score 0 to 3) and the median MRC sum score was 28 (12-38). Cytoalbuminologic dissociation was present in 654% of patients (13 of 20 patients who underwent lumbar puncture). The demyelinating and axonal subtypes each accounted for 47% (9 of 19 patients who underwent an electroneuromyography examination). Immunoglobulins and intravenous corticosteroid therapy were administered in 18% (n=5) and 50% (n=14) of patients, respectively. Lethality rate during hospitalization was 11% (n=3). The median MRC sum score at 6 and 12 month-outcome were 40 (38-49) and 51 (46-58), respectively. After one year of evolution, case fatality rate was 18% (n=5), and 78% of survivors (n=14) could walk without assistance, of which 17% (n=3) were asymptomatic.

**Conclusion:**

In Togo, GBS, with a low hospital prevalence, remains a serious condition due to its high morbidity and lethality related to non-optimal treatment and delayed management.

## Introduction

Le syndrome de Guillain-Barré-Strohl plus connu sous le nom de syndrome de Guillain-Barré (SGB) est une polyradiculonévrite d'installation subaiguë caractérisée typiquement par des troubles sensitivo-moteurs d’évolution ascendante, une dissociation albumino-cytologique dans le liquide cérébrospinal (LCS) et une atteinte démyélinisante à l’électroneuromyogramme (ENMG) [4, 18]. Dans deux tiers des cas, la survenue du SGB est précédée d'un épisode infectieux aigu viral ou bactérien, d'origine respiratoire ou digestive. Le mécanisme physiopathologique actuellement proposé est celui d'un mimétisme moléculaire entre les antigènes des agents pathogènes et les gangliosides présents au niveau du système nerveux périphérique. Le mimétisme antigénique serait alors responsable d'une réaction auto-immune dirigée contre la myéline, l'axone ou les régions paranodales. Le rôle dans la survenue du SGB de certains agents infectieux (Campylobacter jejuni, cytomégalovirus, Epstein-Barr Virus, virus du Zika) et auto-anticorps (Ac anti GQ1b) est établi [8, 12, 22].

En Afrique subsaharienne où on note un plateau technique médical limité, une faible couverture sanitaire et une fréquence relativement plus élevée de maladies infectieuses, il n'existe que quelques études sur le SGB. Le Togo est situé dans une zone intertropicale de l'Afrique de l'Ouest, entre les méridiens 0°20 et 1°50 Est et les parallèles 6° et 11°10 Nord. Sa situation géographique lui confère des climats chauds avec des températures qui varient entre 19, 1 °C et 33 °C. Près de 55% de la population togolaise vit en situation de pauvreté multidimensionnelle. Le salaire minimum interprofessionnel garanti (SMIG) au Togo est de 35 000 F CFA (53 €) alors que le taux de couverture de l'assurance santé est estimé à près de 5% [1]. Nous avons réalisé une étude hospitalière dont l'objectif général était de décrire les éventuelles particularités du SGB dans notre contexte.

## Méthodologie

Le service de neurologie du CHU Campus situé à l'extrême sud du Togo est le centre de référence nationale pour les affections neurologiques. Le seul service de réanimation publique se trouve au CHU Sylvanus Olympio situé à 6 km du CHU Campus de Lomé et le transport n'est généralement pas médicalisé pour les transferts.

Nous avons réalisé une étude de cohorte constituée de patients suivis dans le service de neurologie du CHU Campus de Lomé pour un SGB. Les patients présentant un SGB selon les critères de certitude diagnostique de Brighton des niveaux 1, 2 ou 3 (Tableau I [7]) ont été inclus de façon consécutive. Les patients ont été évalués à l'admission, à la sortie puis à 6 mois et à 1 an d’évolution à l'aide du score d'invalidité SGB (GBS score) ou score de Hugues (Tableau II [21]) et du score neuromusculaire du Medical Research Council (MRC sum-score) (Tableau III [11]).

**Tableau I:** Critères de Brighton pour le diagnostic de syndrome de Guillain-Barré (SGB) [7] Brighton criteria for the diagnosis of Guillain-Barré syndrome [7]

Critères diagnostiques	Niveaux de certitude diagnostique		
	1	2	3
1. Faiblesse motrice bilatérale et flasque aux 4 membres	+	+	+
2. Hyporéflexie ou aréflexie dans les membres atteints	+	+	+
3. Maladie d’évolution monophasique	+	+	+
4. Evolution entre l'installation et le nadir entre 12 heures et 28 jours, suivie d'un plateau	+	+	+
5. Dissociation albumino-cytologique (élévation du taux des protéines et lymphocytes < 50/ mm^3^ dans le liquide céphalo-rachidien)	+	+/#	
6. Altérations électrophysiologiques compatibles avec un SGB	+	#	
7. Absence d'un autre diagnostic expliquant la faiblesse	+	#	+

Niveaux de certitude du diagnostic ayant de 1 (plus probable) à 3 (moins probable): Niveau 1: le niveau le plus élevé- le diagnostic de SGB est plus probable. Tous les items sont positifs Niveau 2: items 1-4 positifs; # 5 (LCR) positif, ou si le LCR n'a pas été effectué/n'est pas disponible: 6 (électroneuromyogramme) et 7 positifs Niveau 3: items 1-4 positifs et 7 positifs

**Tableau II:** Score de Hughes ou échelle de handicap du syndrome de Guillain-Barré [21] Guillain-Barré syndrome disability scale or Hugues score [21]

Cotation	Paramètre de cotation
0	Asymptomatique
1	Symptôme mineur, patient capable de courir
2	Incapable de courir mais capable de marcher plus de 10 m sans aide
3	Capable de marcher mais, sur moins de 10 m et/ou avec aide
4	Incapable de marcher, confiné au lit ou en fauteuil roulant
5	Nécessité d'assistance ventilatoire
6	Décès

**Tableau III:** Score Medical Research Council [11] Medical Research Council sum score [11]

Cotation de la force musculaire	Mouvements évalués
0. Pas de contraction musculaire	Abduction de l’épaule
1. Contraction présente, sans mouvement	Flexion du coude
2. Mouvement après soustraction de gravité	Extension du poignet
3. Mouvement contre gravité	Flexion de la hanche
4. Mouvement contre gravité et contre pression exercée par l'examinateur	Extension du genou
5. Force normale	Flexion dorsale du pied

Chaque groupe musculaire est évalué de 0 à 5 et la somme des scores des groupes musculaires correspond au MRS sum score qui varie de 0 à 60.

Les données ont été saisies et analysées à l'aide du logiciel SPSS version 21. Les variables qualitatives et quantitatives ont été exprimées, respectivement, en fréquence et en médiane avec des intervalles interquartiles de 25 et 75% (IQ).

## Résultats

Entre le 1^er^ mai 2015 et le 31 juillet 2019, 7012 patients dont 3436 femmes (49%) ont été hospitalisés. Parmi eux, 28 (0,4%) présentaient un SGB: certitude diagnostique de niveau 1 chez 17 patients (61%), de niveau 2 chez 8 (28%) et de niveau 3 chez les trois derniers (11%). Des 28 patients inclus dans notre étude, 20 étaient de sexe féminin (71%), soit un sexe ratio de 0, 4. L’âge médian des patients était de 40 (IQ: 27-53) ans.

Les principaux antécédents étaient l'alcoolisme (n=4 soit 14%), l'infection à VIH (n=4 soit 14%), la bronchopneumopathie chronique obstructive (n=3 soit 11%) et l'hypertension artérielle (n=3 soit 11%). Aucun des patients VIH positifs n’était au stade sida. Chez 10 de nos patients (36%), aucun antécédent de pathologie médicale n’était retrouvé.

Chez 18 patients (65%), un évènement ayant précédé les symptômes neurologiques était noté dans un délai médian de 14 (IQ: 7-18) jours: épisode fébrile non étiqueté (n=13 soit 46%), gastroentérite (n=7 soit 25%), rhinite (n=5 soit 18%) et stress intense (n=1 soit 4%).

Le motif de consultation le plus fréquent était la sensation de lourdeur des membres inférieurs (n=24 soit 86%).

Les paresthésies et la difficulté à déglutir étaient rapportées respectivement dans 11% (n=3) et 4% (n=1) des cas.

Le délai médian de la première consultation indépendamment de la structure d'accueil était de 2 (IQ: 2-3) jours. Les médecins généralistes étaient les praticiens le plus souvent consultés en premier recours. Pour 71% (n=20) des patients, l'origine de la maladie était mystique et 40% (n=11) des patients s’étaient adressés en première intention à un prêtre, un pasteur ou à un guérisseur. Le délai médian de consultation auprès d'un neurologue était de 4 (IQ: 3-8) jours.

**Figure 1:**
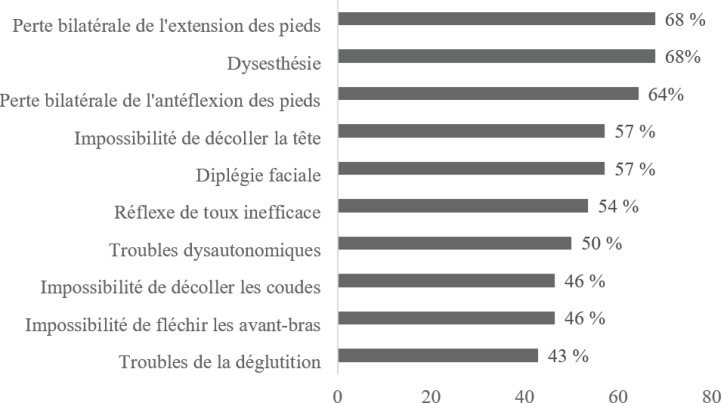
Troubles neurologiques observés à l'admission chez 28 patients souffrant d'un syndrome de Guillain-Barré Neurological disorders observed at admission in 28 patients with Guillain-Barré syndrome

À l'admission, la marche était possible chez 39% (n=11) des patients (GBS score 0 à 3); 48% (n=13) et 17% (n=5) des patients avaient un GBS score respectivement à 4 (nécessité de fauteuil) et à 5 (nécessité d'assistance ventilatoire); le MRC sum score médian était à 28 (IQ: 12-38). La figure 1 présente les différents troubles neurologiques à l'admission: perte bilatérale de l'extension des pieds avec aréflexie ostéotendineuse et paresthésies chez 70% des patients (n=20), diplégie faciale (54%, n=15), troubles dysautonomiques (50%, n=14) dont accès de tachycardie (43%, n=12), labilité tensionnelle (36%, n=10), iléus paralytique (25%, n=7) et rétention aiguë d'urine (18%, n=5). Les douleurs neuropathiques étaient rapportées par 43% des patients (n=12).

La ponction lombaire (PL) a été réalisée chez 20 patients (71%). Le délai médian de réalisation de la PL par rapport au début des symptômes neurologiques était de 9 (IQ: 6, 3-13) jours. La dissociation albumino-cytologique était observée chez 13 patients (65%). La protéinorachie médiane était de 2, 4 (IQ: 1-3, 5) g/l. La cytorachie médiane était de 0, 5 (IQ: 0-3, 75) cellules/mm3.

L'ENMG a été réalisée chez 19 patients (68%). Le délai médian de réalisation de l'ENMG par rapport au début des symptômes neurologiques était de 15 (IQ: 7-18) jours. Les formes démyélinisantes (acute inflammatory demyelinating polyneuropathy, AIDP) représentaient 47% (n=9), les formes axonales motrices pures (Acute Motor Axonal Neuropathy, AMAN) 47% (n=9) et la forme inexcitable 6% (n=1).

À la phase aiguë, les patients dès leur admission ont été considérés comme une urgence neurologique avec surveillance régulière notamment de la tension artérielle, des performances respiratoires (comptage en apnée) et de l’état neurologique. Un seul patient a été pris en charge dans le service de réanimation. Parmi les 28 patients, 5 (18%) ont bénéficié d'immunoglobulines par voie intraveineuse (Ig IV) à raison de 0, 4 g/kg/j pendant 3 jours dans un délai médian de 14 (IQ: 9, 5-22, 5) jours. Quatorze patients (50%) ont bénéficié d'une corticothérapie par voie intraveineuse (2 mg/kg/jour pendant 5 jours). Neuf patients (32%) n'ont reçu aucun traitement.

Parmi les 28 patients, 10 soit 36% ont nécessité un traitement à visée respiratoire: oxygénothérapie par lunettes dans 8 cas (29%) et ventilation assistée dans 2 cas (7%). Un des deux patients intubés a eu une trachéotomie. Tous les patients ont reçu une prévention de la maladie thromboembolique par héparine de bas poids moléculaire.

À la phase subaiguë, la rééducation fonctionnelle s'est résumée à la poursuite à domicile de la kinésithérapie motrice débutée au cours de l'hospitalisation.

L’évolution était monophasique chez tous les patients. La durée médiane d'hospitalisation était de 12 (IQ: 7-18) jours. En cours d'hospitalisation, trois patients (11%) sont décédés par détresse respiratoire sévère respectivement 2, 14 et 15 jours après l'admission. Deux d'entre eux étaient traités par corticothérapie. À la sortie de l'hospitalisation, l'absence de douleurs était notée chez 9/11 patients (82%) ayant reçu la corticothérapie contre 5/7 (71%) ne l'ayant pas reçue (p=0, 14). Après le retour à domicile, deux patients sont décédés, l'un à 35 jours et le second à 76 jours d’évolution, portant le taux de létalité à 18% à un an. Une embolie pulmonaire a été la cause évoquée pour ces décès. Chez les 18 survivants, le MRC sum score médian était de 40 (IQ: 38-49) à 6 mois et de 51 (IQ: 4-58, 5) à un an d’évolution. Le GBS score 0 à 3 était obtenu à 6 mois chez 15/18 patients (83%) et à un an, chez tous les 18 patients dont 14 pouvaient marcher sans aide et 3 asymptomatiques. Les principales séquelles à un an d’évolution étaient un steppage bilatéral (11/15 patients), une diparésie faciale (4/15), une hypophonie (4/15), des douleurs neuropathiques (2/15) et une amyotrophie (jambiers antérieurs: 12/15, éminences thénars/hypothénar: 12/15). La récupération du groupe ”AIDP” n’était pas significativement différente de celle du groupe ”AMAN”. Le tableau IV résume les caractéristiques des patients à 6 mois et à un 1 an d’évolution comparativement à celles de l'admission.

**Tableau IV:** Caractéristiques des patients souffrant de syndrome de Guillain-Barré à l'admission, à 6 mois et à un an d’évolution (n=28) Characteristics of patients with Guillain-Barré syndrome at admission, at 6 months and at 1 year of evolution (n=28)

GBS Score	Admission	6 mois	1 an
0 = Asymptomatique	0% (n=0)	0% (n=0)	18% (n=5)
1 = Symptômes mineurs	4% (n=1)	0% (n=0)	12% (n=3)
2 = Marche sans aide	0% (n=0)	22% (n=6)	47% (n=13)
3 = Marche avec aide	31% (n=9)	43% (n=12)	6% (n=0)
4 = Fauteuil	48% (n=13)	17% (n=5)	0% (n=0)
5 = Nécessité d'assistance ventilatoire	17% (n=5)	0% (n=0)	0% (n=0)
6 = Décès	0% (n=0)	18% (n=5)	18% (n=5)
Douleurs Neuropathiques*	43% (n=12)	11%* (n=3)	11%* (n=3)
MRC sum score médian (intervalle interquartile)	28 (12-38)	40 (38-49)*	51 (46-48)*

* Données pour 23 patients, 5 patients n'ayant pas pu être examinés à 6 mois et un an d’évolution

## Discussion

Nous rapportons les caractéristiques cliniques et évolutives du SGB dans notre contexte de pays à ressources limitées à travers une étude de cohorte suivie pendant un an. Les études dans les conditions similaires où l'accès aux soins est limité, sont peu nombreuses [2, 3, 17] (Tableau V). Le caractère monocentrique et la taille réduite de l’échantillon de l’étude en constituent les principales limites. L'insuffisance du plateau technique rend la majorité des diagnostics étiologiques plus probables que d'autres.

**Tableau V:** Séries de cas sur le syndrome de Guillain-Barré en Afrique de l'Ouest Comparison of West African cases series on Guillain-Barré syndrome

	Osuntokun [17] (Nigéria)	Basse et al [4] (Sénégal)	Apetse et al [cette étude] (Togo)
**Taille de la série Période d’étude**	34 nigérians 1960-1971	39 sénégalais 2010 et 2016	28 togolais entre 2015 et 2019
**Age (ans)**	Tranche médiane [20-29] ans	Moyen: 33, 9	Médian: 40 (27-53)
**Sexe ratio**	1, 42	0, 69	0, 40
**Délai début des symptômes-prise en charge**	Moins de 3 semaines	Non disponible	Médian: 4 (3-8) jours
**Atteinte respiratoire**	2,9%	Non disponible	36%
**Formes axonales**	29,4%	24%	47%
**Taux de mortalité**	5,9% à 3 mois	10,2% à 3 mois	18% à 6 mois

Dans notre série, avec une incidence hospitalière moyenne de 7 cas par an, le SGB est une affection rare ne représentant que 0,39% des patients hospitalisés en neurologie. La présentation clinique est classique avec des troubles sensitivo-moteurs bilatéraux des membres à prédominance crurale associées à une diplégie faciale et précédés d'un évènement infectieux. À l'admission, 14% des patients nécessitaient une prise en charge respiratoire. Sur le plan paraclinique, la classique dissociation albumino cytologique était retrouvée dans 64% des cas dans un délai médian de réalisation de la PL par rapport au début des symptômes neurologiques de 9 (IQ: 6, 3-13) jours. Ces données sont proches de celles rapportées en général [8] et en particulier en Afrique de l'Ouest [3, 17].

Dans cette série, le sex-ratio H/F de 0, 40 est inversé par rapport à ce qui est généralement décrit. Ceci pourrait être expliqué par le fait que devant la même maladie, les hommes ont moins recours aux soins de santé que les femmes.

Les antécédents des patients sont marqués par une importante proportion de l'infection à VIH. L'induction du SGB par le VIH lui-même plutôt que par une infection opportuniste peut être suggérée par l'absence de phase sida chez ces patients. En attendant une étude pour établir le lien exact entre l'infection VIH et le SGB, il convient de suggérer la recherche de l'infection à VIH devant tout SGB dans notre contexte.

Le délai médian entre la consultation spécialisée et le début des troubles de 4 (IQ: 3-8) jours est long. L'errance diagnostique liée à la méconnaissance de l'affection représente un facteur qui pourrait expliquer ce long délai. Une sensibilisation des médecins généralistes, premiers recours médicaux pourrait aider à diminuer ce délai. Le fait que 71% des patients évoquent une origine mystique au SGB constitue un autre aspect à prendre en compte car les perceptions collectives de la maladie en Afrique s'orientent vers une causalité exogène, endogène ou sociégène comme le salaire du péché, l'envoûtement… [5, 15]. Ceci explique le recours en premier lieu aux prêtres, pasteurs ou guérisseurs.

Parmi les 68% des patients ayant réalisé l'ENMG, les formes motrices axonales pures étaient tout aussi fréquentes que les formes démyélinisantes. Dans la série de Basse et al [3] au Sénégal, la forme AMAN n'a représenté que 24% des cas de SGB. La répartition des SGB selon les sous–types ENMG varie d'une région à l'autre voire au sein d'un même pays [6, 8, 16, 20]. Les causes de ces différences restent à identifier. L’étude des anticorps associés au SGB dans notre contexte permettrait d'identifier les éventuels agents infectieux inducteurs du SGB en vue d'une prévention primaire.

Les traitements immuno-modulateurs d'efficacité prouvée dans le cas des SGB sont les échanges plasmatiques (EP) et les Ig IV: 4 à 5 séances d'EP ou 2 g/kg d'Ig IV pendant 3 jours de façon optimale dans les deux premières semaines [9, 13, 19]. La corticothérapie orale ou intraveineuse, en dehors de son intérêt décrit par certains auteurs dans la prise en charge des douleurs neuropathiques au cours du SGB, qu'elle soit utilisée seule ou en association aux Ig IV ou aux EP n'ont pas fait la preuve de leur efficacité [10, 14, 19]. Au Togo, le coût de l'ENMG est entre 40 000 et 60 000 F CFA (61 et 91 € respectivement). Les Ig IV coûtent environ 250 000 F CFA/gramme (381 €). En raison de la non-disponibilité des EP et du coût élevé des Ig IV, la prise en charge se résume à un traitement symptomatique et la corticothérapie dans la majorité des cas. Dans notre série, l'administration des corticoïdes n'a pas amélioré les douleurs à la sortie. Cette étude ne conforte donc pas l'intérêt de la corticothérapie à visée antalgique dans le SGB. L’évolution dans notre étude a été caractérisée par des taux de létalité hospitalière et globale respectivement de 11% et 18%. Ces taux de létalité sont proches des 10% dans la série de Basse et al [4] au Sénégal. Ils pourraient être réduits par la détection rapide du SGB, la prévention et la prise en charge efficace des complications respiratoires. De la relative faible proportion de patients ayant récupéré la marche sans aide à un an d’évolution ressort la nécessité du renforcement de nos structures de réadaptation fonctionnelle.

## Conclusion

Au Togo, le SGB est caractérisé par une prévalence hospitalière faible, une prédominance féminine et une présentation clinique classique. Le sous-type axonal est tout aussi fréquent que le sous-type démyélinisant. La croyance en l'origine mystique du SGB rend compte de l'importante proportion de patients ayant un premier recours autre que l'hôpital. Le SGB, dans notre contexte reste une affection grave de par sa forte morbi-létalité en lien avec des thérapeutiques non optimales mais aussi avec le retard de consultation spécialisée. Pour réduire cette morbi-létalité, une sensibilisation des médecins généralistes pour une meilleure connaissance du SGB, une prévention et une détection rapide des complications respiratoires ainsi qu'un renforcement des structures de réadaptation paraissent nécessaires.

## Conflits D'intérêts

Les auteurs ne déclarent aucun conflit d'intérêt.
